# Effects of repetitive low-acceleration impacts on attitude estimation with micro-electromechanical inertial measurement units

**DOI:** 10.3389/frobt.2023.1211531

**Published:** 2023-08-23

**Authors:** Federico Allione, Juan D. Gamba, Antonios E. Gkikakis, Roy Featherstone, Darwin Caldwell

**Affiliations:** ^1^ Department of Advanced Robotics (ADVR), Istituto Italiano di Tecnologia, Genoa, Italy; ^2^ Department of Informatics, Bioengineering, Robotics and Systems Engineering (DIBRIS), University of Genoa, Genoa, Italy

**Keywords:** IMU, MEMS, orientation estimation, drift, legged robots, failure detection and recovery

## Abstract

Inertial Measurement Units are present in several applications in aerospace, unmanned vehicle navigation, legged robots, and human motion tracking systems, due to their ability to estimate a body’s acceleration, orientation and angular rate. In contrast to rovers and drones, legged locomotion involves repeated impacts between the feet and the ground, and rapid locomotion (e.g., running) involves alternating stance and flight phases, resulting in substantial oscillations in vertical acceleration. The aim of this research is to investigate the effects of periodic low-acceleration impacts (4 g, 8 g and 16 g), which imitate the vertical motion of a running robot, on the attitude estimation of multiple Micro-Electromechanical Systems IMUs. The results reveal the presence of a significant drift in the attitude estimation of the sensors, which can provide important information during the design process of a robot (sensor selection), or during the control phase (e.g., the system will know that after a series of impacts the attitude estimations will be inaccurate).

## 1 Introduction

As legged robots move from the laboratory into our daily lives, there is an expectation that their motor skills will improve. In particular, they should be able to walk, run, jump, and so on, at speeds comparable to those of humans and animals (Semini et al., 2011; Hutter et al., 2016; Semini et al., 2017; Katz et al., 2019). This places significant demands on their actuators, controls and mechanical parts, but also on their sensors.

Micro-Electromechanical Systems (MEMS), such as Inertial Measurement Units (IMU), are embedded in almost all mobile robots thanks to the variety of measured signals they provide. They contain on-board processing systems called Attitude and Heading Reference Systems (AHRS) which combine the output of several sensors, such as a 3-axis accelerometer, a 3-axis magnetometer, and a 3-axis gyroscope to estimate a system’s attitude, using a variety of algorithms, the details of which are usually proprietary. One particular computed output is the robot’s orientation, which must be accurate up to a certain degree for the robot to operateas expected. Inaccurate estimation of orientation can cause a robot to veer off course or lose its balance and fall. In this paper, we refer to these systems generically as IMU because it is in this way that the larger audience usually calls them.

This paper investigates the effect on a selection of IMUs of substantial oscillations in vertical acceleration caused by the alternation between flight phase and stance phase during fast legged locomotion. It does this using a “bounce test” apparatus, as described in Section 3. We call these oscillations “low-acceleration impacts” in order to distinguish them from the much higher accelerations experienced during accidental collisions and falls. The motivation for this research is the lack of claims, both in the manufacturers’ literature and the research literature, that these devices have been designed and tested for use under these conditions.

Specifically, the experiments reported in this paper investigate the following items.

1) Drift in the IMU’s orientation estimates during approximately vertical bouncing motion;
2) The timescale on which this drift occurs; and
3) The time it takes the IMU to recover after bouncing ceases.

The results show significant drift in all three IMUs tested, including drift that exceeds the maximum error stated in the datasheet.

Even though several legged robots have already demonstrated motions involving substantial repetitive vertical accelerations Raibert (1986); Katz et al. (2019); Yim et al. (2020), the effects of these motions on IMUs have not been properly investigated. To achieve consistent and reliable behaviours, legged robots must be aware of their electronics’ limitations. This issue can be addressed at the design stage by choosing an IMU that is accurate enough under the expected operating conditions, or it can be addressed at the control stage by adapting the robot’s motion planning and control to make allowance for the IMU’s limitations. Either way, the first step is to identify and measure those limitations. Inadequately addressing these limitations could lead to the emergence of uncontrollable robots. For instance, in Allione et al. (2022), a balancing machine relies on an IMU to estimate orientation and maintain balance. However, if the sensor exhibits inconsistent behaviour, it could lead to unrecoverable states.

The rest of the article is organized as follows. Firstly, a background in IMU drift estimation is presented. Then, the experimental setup is described together with the experimental process. Afterwards, the results are presented and discussed, followed by the conclusions and the acknowledgements.

## 2 Background

The accuracy of MEMS sensors for state estimation during dynamic behaviours has been previously studied but under different working conditions to those considered in this work.

Position estimation using IMUs has been addressed by Zihajehzadeh et al. (2015), where the authors propose a cascaded Kalman filter with a fusion of GPS and IMU data for trajectory tracking. In humanoid applications, high-quality measurement of the floating base orientation can be achieved with an IMU, but achieving high-precision positioning with low drift remains a significant challenge. Kuindersma et al. (2015) describe different optimization strategies and a state estimation algorithm to execute walking over non-flat terrains with the Atlas^1^ humanoid. The authors used an IMU mounted on the pelvis to obtain its pose and twist. To reduce the drift of the robot measurements, they used an inertial and kinematic estimator, which was proven to be unsuitable for accurate walking over distances of tens of meters. They finally added a LIDAR (Laser Imaging, Detection, And Ranging) to achieve the task. Instead, Liang et al. (2018) analyze the effects of IMU drift on a walking person by using an IMU to estimate the vertical motion of the foot. However, the experiment is relatively short since the subject takes only a few steps. Abdulrahim et al. (2010) use a MEMS IMU to estimate the position of a walking person in an indoor environment where no GPS signal can be used. It calculates the body’s location while moving, but the precision required is not the same as the one required by most robot-control algorithms.

On the topic of orientation estimation, in Safaeifar and Nahvi (2015), the authors attempt to reduce the effect of drift in orientation tracking for a virtual reality head-mounted display. The sensor is mounted directly on the headset, and it experiences continuous motion but with very low accelerations. Li et al. (2014) present a three-dimensional model of a quadruped robot with six degrees of freedom at the torso and five degrees of freedom at each leg executing a 3D trotting gait. The IMU (mounted on the robot’s torso) experiences a severe drift on the yaw signal during these experiments. The authors believe that this drift was caused by the magnetic field excited by the motor in the treadmill. Zihajehzadeh et al. (2014) propose a cascaded two-step Kalman filter to compensate for external ferromagnetic disturbance or large impulsive acceleration (such as a single jump) or medium-long acceleration (roller-blade outdoors). Tan et al. (2021) avoid the usage of magnetometers to estimate the foot progression angle by means of IMU data. However, the experiments are limited to only a few steps and only to people walking. The effects of continuous low amplitude accelerations (e.g., a person walking) on orientation estimation have been addressed by Cardarelli et al. (2020). In this work, the authors collect accelerometer and gyroscope raw data of people walking on a treadmill, and then they process those data offline by means of a custom filter to compensate for drift and estimate the correct orientation. Such a strategy is often not feasible for embedded systems, which typically lack memory and computational power and must rely on the orientation data provided directly by the sensors.

IMUs have gained significant popularity in the field of wearable devices. Yang et al. (2018) combine an air-pressure and IMU sensor to recognise human activities, while Qi and Aliverti (2020) use a chest-worn band equipped with a breathing sensor and tri-axis accelerometer to measure respiratory parameters and identify human activities. A comprehensive literature review conducted by Arlotti et al. (2022) focuses on IMU-based wearable devices in sports medicine. The review concludes that the current limitations of IMU drift and challenging calibrations are limiting the transition of these devices from research laboratories to the commercial market.

## 3 Experimental setup

**FIGURE 1:**
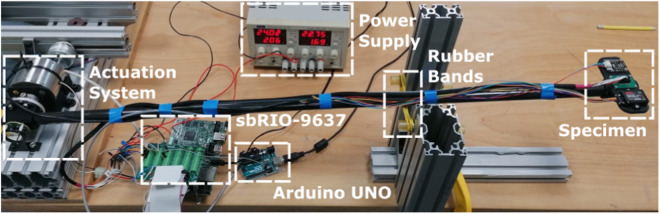
Experimental setup.

**FIGURE 2:**
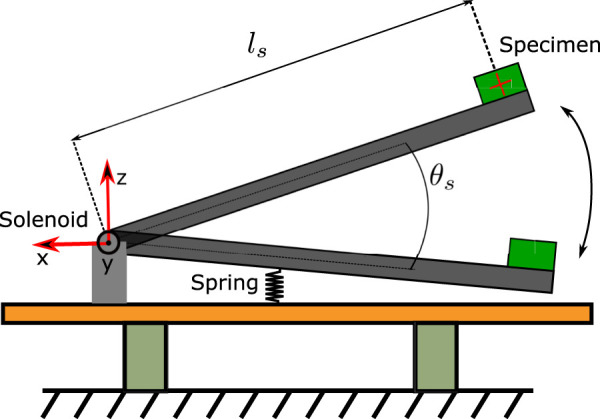
Qualitative representation of the experimental setup. The spring is made with rubber bands.

The experiment investigates the effects of prolonged, continuous vertical bouncing motion on the accuracy of orientation estimation on a selection of MEMS IMUs. The apparatus is shown in Figures 1, 2, and consists of a long rod with a rotary actuator at one end and the IMUs at the other. Strictly speaking, this means that the IMUs travel along a circular arc. However, the radius of the circle is large enough for it to be a reasonable approximation to vertical motion. This section is divided into three subsections describing the actuation system, the specimen and the experiment itself.

### 3.1 Actuation system

The actuation system consists of a Magnet Schultz^2^ Proportional Rotary Solenoid Type GDR075X20A61-S1 powered by a Pololu^3^ G2 High-Power Motor Driver. This solenoid produces pure rotational motion and offers controllable bidirectional torque, making it easier to measure the rotation angle and control it compared to most of the other so-called rotary solenoids, which produce helical motion and do not allow controllable bidirectional torque. The solenoid directly actuates one end of a 1 m carbon fiber tube of 20 mm external diameter and 0.5 mm wall thickness. On the other end of the tube, opposite to the solenoid, the tested specimen is placed. The angular position of the tube is measured with an AksIM-2^4^ absolute position encoder, sampled at 500 Hz by the National Instrument Single Board sbRIO-9637 FPG^5^, which also generates control signals for the Pololu driver. The sampling frequency has been chosen to match the one used to sample both the VN100 and the 3DM-GX5-15 IMUs. The spring shown in Figure 2 is physically realized by means of rubber bands.

### 3.2 Specimen

**FIGURE 3:**
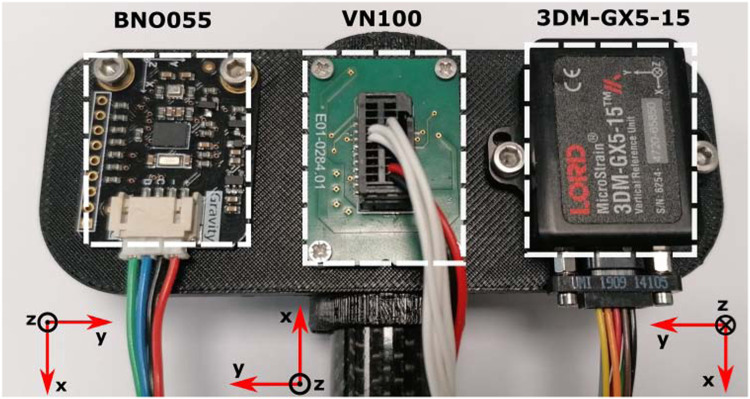
Top view of the IMUs mounted at the end of the carbon fiber rod and their reference system. The perspective distortion in this photo makes the BNO055 and 3DM-GX5-15 appear to be at an angle, when in reality they are aligned.

The specimen consists of three independent IMUs provided by three different vendors; each IMU has a different communication interface. They are mounted on a custom-made 3D-printed support to allow them to have the *y*-axis aligned with the rotation axis of the solenoid (see Figure 3).

#### 3.2.1 VN100

The Vectornav VN100^6^ is the IMU that can measure the highest linear acceleration (up to 16 g). It is sampled at 500 Hz, which is the maximum orientation estimate update rate configurable for the device, by the sbRIO-9637 board using the SPI communication protocol. The IMU chip is mounted on a custom-made PCB, and is intended to be used in a highly athletic monopedal robot called Skippy (Allione et al., 2021; Gkikakis, 2021), which is designed to perform repetitive vertical hops, among other behaviours.

#### 3.2.2 3DM-GX5-15

The Lord MicroStrain 3DM-GX5-15^7^ can measure linear accelerations up to 8 g. It communicates through a USB interface with the software SensorConnect^8^ provided by the vendor. The IMU is configured to stream data at 500 Hz continuously and it is directly connected to the host PC using a USB cable. The 3DM-GX5-15 is the only tested IMU that is not equipped with a magnetometer.

#### 3.2.3 BNO055

The Bosch BNO055^9^ is assembled on a DFRobot breakout^10^. Although the DFRobot breakout is discontinued, the BNO055 chip can be found in other breakouts with identical characteristics provided by other vendors, such as Adafruit^11^. It is the cheapest among the tested IMUs (it can be bought for just a few tens of euros). The BNO055 provides orientation data only when configured in ‘fusion mode’; such a configuration provides a 4 g linear acceleration range and an output data rate of 100 Hz. The BNO055 does not have any non-volatile memory to store the calibration data, hence every time it is powered on it has to be calibrated. The gyroscope is calibrated by holding the IMU still for some seconds, and moving manually the rod up and down proved to be sufficient to calibrate the accelerometer. The magnetometer, instead, requires a 3D change in the orientation of the device to get calibrated. Such movements are not compatible with our setup and as a consequence the IMU is never fully calibrated. An Arduino UNO board^12^ samples the device at 100 Hz by using the I2C communication protocol. This selection is used to compare professional and expensive acquisition systems with a hobbyist and cheap one.

### 3.3 Experiment description

**FIGURE 4:**
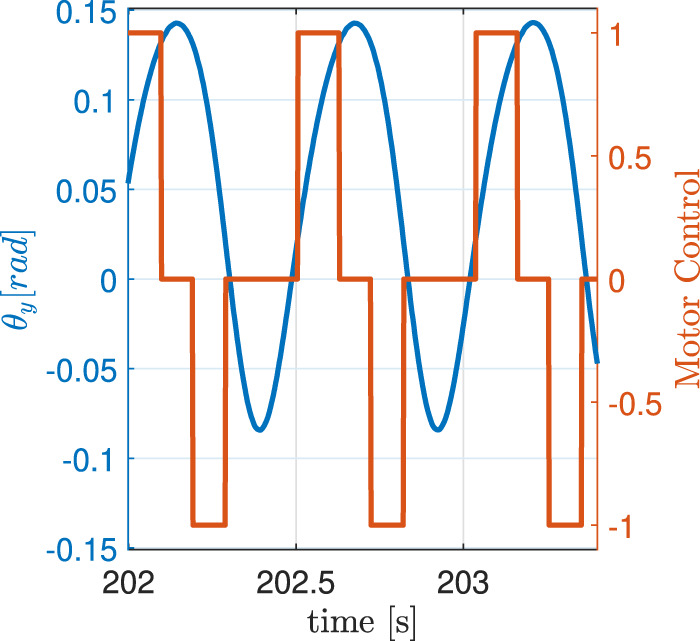
Motor control signal with respect to the angular position of the tube during the continuous bouncing of the 4 g experiment. The torque is applied only in the direction of motion and when the tube is above the rubber bands and below the bounce apex. The zero line represents the rest position of the rod.

The experiment consists of continuously bouncing the tube against the rubber bands, which act as a spring and push the rod back up. The solenoid’s torque (which is small compared to the forces exerted by gravity and the rubber bands) is actuated with a constant voltage in the same direction as the rod’s motion, and it is activated only when the rod’s angle, measured by the encoder, is within a specified range (Figure 4). The lower bound of this range is when the rod is about to touch the rubber bands, and the upper bound depends on the desired maximum acceleration at the bounce: the higher the bounce, the higher the acceleration. The system is tuned to have the bounce apex always above the upper threshold of the applied torque range. The strategy of applying a constant torque over a constant (angular) stroke implies a constant energy injection per bounce, so that the apparatus will converge and settle to a steady state in which the bounce height is such that energy losses match energy inputs. The idea is to have a smooth transition between the stance and flight phases, which are the two components of the continuous bouncing behaviour of a hopping robot.

The experiment starts by turning on the apparatus, then a sufficient amount of time is allowed to let all the sensors power on correctly. At this point, the rod is manually moved up and down until the BNO055 is calibrated. The following phase starts by logging the sensors with the rod at rest position for about 1 minute. The data from all the IMUs are recorded. Then, the rod is manually pushed downwards against the rubber bands to start the bouncing motion (Figures 6A, 7A, 8A) which continues for about 3 minutes. Finally, the solenoid is switched off, so that the bouncing ceases, and the system continues logging the sensors for another minute with the rod remaining still at its rest position (Figures 6B, 7B, 8B). The data collected from the IMUs are the compensated linear accelerations, the compensated angular velocities and the quaternions. The measured quaternions are then converted offline into Euler angles allowing the rotation around the *y*-axis of the IMUs to be compared with the angular position of the tube. The aim of the experiment is to discover the magnitude of drift in the absolute orientation of the IMUs while continuously bouncing, the time it takes for this drift to emerge, and the time required to recover afterwards.

The experiment consists of three parts with increasing linear acceleration values. For each acceleration value, the investigation is performed three times. Each IMU is tested at an acceleration that is close to, but strictly below, its accelerometer’s saturation limit, and the collected data is checked at the end of the experiment to make sure that the accelerometer never saturated. Any trial in which the accelerometer reached its saturation limit is rejected and replaced with a new one.

Since the three IMUs have the *z*-axis vertical, the experiments are classified based on the maximum acceleration on the *z*-axis (4, 8 and 16 g). 4 and 8 g accelerations are comparable to those experienced by the human ankle during running at the moment when the foot touches the ground (Table I of Koga et al. (2020); Figure 7 of Bhattacharya et al. (1980)).

Table 1 reports the accuracy for each IMU and for which experiments they have been tested. The different bouncing acceleration values are obtained by changing the rubber bands’ distance from the axis of rotation of the tube or increasing the actuated range of motion of the solenoid, or varying the number of rubber bands.

**TABLE 1:** IMUs accuracy. Angles are expressed both in degrees [deg ] (left) and radians [*rad*] (right). The last column reports the experiments in which each IMU was tested. Short dashes denote data that are not provided by the manufacturer.

IMU	Roll/Pitch		Yaw		Experiments
BNO055	—	—	—	—	4 g
3DM-GX5-15	0.25	0.0044	—	—	4, 8 g
VN100	1.00	0.0175	2.0	0.0350	4, 8, 16 g

## 4 Data acquisition system

Each IMU has a different communication interface, and therefore it requires its own independent data acquisition system; the three processes are synchronized with a dedicated software interface running on the host PC.

A different delay in the received data from each IMU is observed due to multiple interfaces and acquisition systems. As a consequence, all the recorded data are synchronized offline once the experiment is completed before analyzing the results.

### 4.1 National instrument sbRIO-9637

The National Instrument sbRIO-9637 board has analog and digital inputs/outputs, a dual-core CPU and a programmable FPGA. The board is configured based on a Supervisory Control and Data Acquisition (SCADA) architecture, where the FPGA oversees performing the SPI communication and controlling the solenoid. The sbRIO-9637 is used to sample the position encoder and the VN100 using two different SPI channels, both of them at 500 Hz. The board’s CPU uses these measurements to control the solenoid and sends them to the host using network streams. Finally, the host runs a Human-Machine-Interface (HMI) to monitor online the acquired data.

### 4.2 Arduino UNO

The Arduino UNO board is the acquisition system for the Bosch BNO055. The board communicates with the IMU with an I2C interface; it samples the data at 100 Hz and then it sends them to the PC through a serial interface.

### 4.3 Host computer

The data acquisition system for the Lord MicroStrain 3DM-GX5-15 is the software SensorConnect provided by the vendor. The sensor is sampled at 500 Hz.

The three independent data acquisition systems are activated simultaneously by a LabVIEW Virtual Instrument (VI). When the start button is pressed in the host’s VI, the sbRIO-9637 FPGA starts logging the VN100 and sets to high a digital output connected to the Arduino Uno to start sampling. The signal returns to low when the user interrupts the logging at the host’s VI. The host’s VI also provides the logging timestamp used in the SensorConnect software to save the captured data of the appropriate time interval.

## 5 Results

The main objective of this work is to investigate how the estimation of the absolute orientation is affected by continuous low-intensity impacts, simulating the shocks that a running/hopping robot may experience. The experimental apparatus is designed to have the *y*-axis of the tested IMUs aligned with the rotation axis of the solenoid, whose angle is directly measured with the absolute position encoder. The physical imperfections of the system mean that each IMU’s *y*-axis is not perfectly aligned with that of the solenoid. To compensate for any possible misalignment, the conversion of the quaternions into Euler angles is performed in a coordinate system that is as close as possible to the IMU’s internal coordinate system but has its *y*-axis accurately aligned with that of the solenoid. The transform from internal to aligned coordinate system is obtained separately for each IMU using its motion data from the first few bounces. The measured angular position of the encoder, $\theta_{y_{e}}$ , and the corresponding angle measured by the IMUs, $\theta_{y_{I}}$ , are referred as *θ*_*y*_ when mentioned together. In order to have comparable measurements, the average of the measured values with the rod in its rest position is defined to be the reference orientation for all the IMUs and both the encoder angle and the IMU orientation estimates are measured relative to this orientation.

One of the outcomes of this experiment is the difference between the angular position measured by the encoder and the angular rotation about the *y*-axis estimated by the IMUs. This value, *E*_Pitch_, will be referred to as orientation error or simply error. During the experiments, the rod presents a minor bending while it presses into the rubber bands. This bending behaviour causes a slightly bigger rotation at the IMUs compared with encoder measurement (see the responses of the IMUs in Figures 6A, 7A, 8A). The error in the two remaining rotation axes (referred to as *E*_Roll_ about the x-axis and *E*_Yaw_ about the *z*-axis) is the difference between the average value of the orientation during the initial rest period, and current orientations, since the sensors are not expected to move about those axes during the experiment. The continuous bouncing of the specimen causes a periodic component (at around 1–2 Hz) in *E*_Roll_, *E*_Pitch_ and *E*_Yaw_. This component has been removed from the error graphs by a 6^th^ order zero-phase low pass filter consisting of a Butterworth filter with a cut-off frequency of 0.2 Hz.

The results of the three experiments are discussed in the following paragraphs. The solid horizontal lines (where present) indicate the accuracy of the IMU as stated in the datasheets provided by the manufacturers. Each experiment is performed three times because having three sets of data allows one to get an approximate idea of how much of the measured signal is random and how much is a repeatable effect caused (we assume) by the disturbing effect of the bouncing motion on each IMU. Table 2 reports the maximum and the average peak acceleration experienced by each IMU to show that none of the IMUs saturated during the experiment. The average linear velocities of the IMUs at landing during the 4, 8 and 16 g experiments are 1.9, 3.5 and 5.8 m/s, respectively. These correspond to drop heights of 0.18, 0.62 and 1.71 m, respectively. However, the actual drop heights of the IMUs in the bounce tester are smaller because the IMUs follow a circular arc and are accelerated by the solenoid as well as gravity. These figures suggest that a legged robot would not experience accelerations as high as 16 g during normal locomotion. However, it should be understood that the rubber bands give the rod a soft landing, and a stiffer landing would produce a larger acceleration for a given velocity. Such accelerations can arise if the robot is mechanically stiff and/or pounds the ground with its feet. This type of motion causes acceleration spikes when the (relatively hard) foot strikes the (hard) ground due to mechanical shock propagating up the leg into the torso, but this effect is absent in the bounce test experiments.

**TABLE 2:** Every experiment is performed three times. For each experiment trial, the table reports the highest recorded value of the linear acceleration on the *z*-axis (left column) and the average value of the peak acceleration on each bounce (right column) on the *z*-axis. The maximum linear acceleration is reached when the specimen reaches the bottom of the bounce and is about to be pushed back by the rubber bands. The saturation values for the three IMUs are measured experimentally and are 155.84 for the VN100, 79.66 for the 3DM-GX5-15 and 39.22 for the BNO055. All the acceleration values are expressed in [m/s^2^].

Experiment						
IMU	First Trial		Second Trial		Third Trial	
4 g						
VN100	39.42	38.76	39.02	38.68	38.81	38.13
3DM-GX5-15	39.47	38.79	39.23	38.83	39.10	38.21
BNO055	38.37	37.88	38.24	37.65	37.98	37.27

8 g						
VN100	76.64	73.83	75.54	74.07	76.56	75.41
3DM-GX5-15	77.71	75.96	76.78	74.87	77.11	75.86
BNO055	—	—	—	—	—	—

16 g						
VN100	153.84	153.80	153.86	153.80	153.85	152.95
3DM-GX5-15	—	—	—	—	—	—
BNO055	—	—	—	—	—	—

**FIGURE 5:**
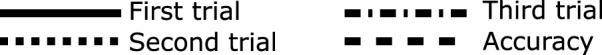
The picture explains the meanings of the different line styles used in the error graphs for *E*_Roll_, *E*_Pitch_ and *E*_Yaw_. The legends in those graphs identify each IMU with a different color. The accuracy line, when present, shows the datasheet figure for maximum error so that it can easily be seen whether or not the actual error exceeds the datasheet figure.

**FIGURE 6:**
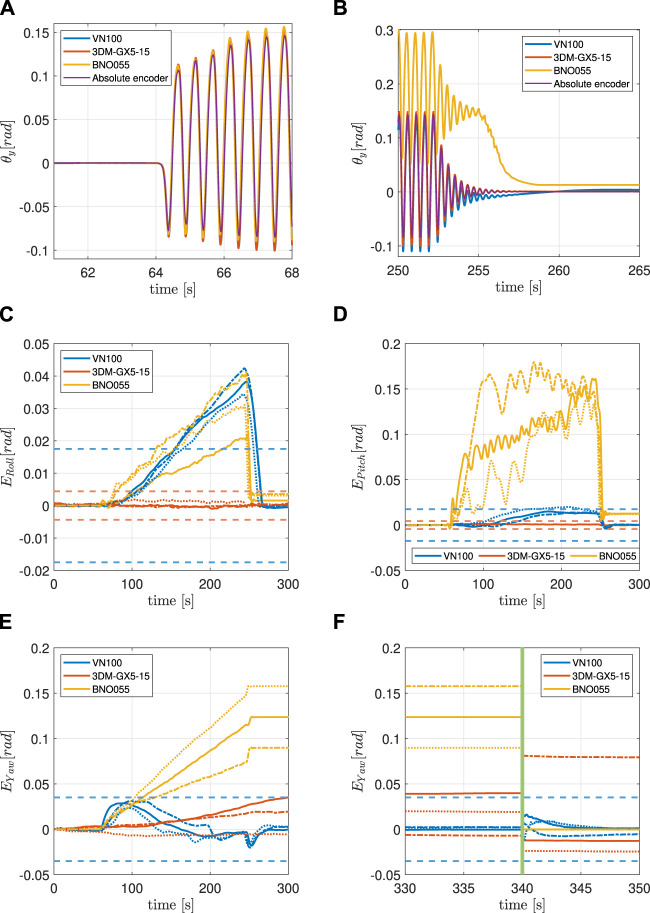
**(A)** IMU and encoder synchronized pitch measurements at the beginning of the 4 g experiment. **(B)** IMU and encoder synchronized pitch measurements at the end of the 4 g experiment. **(C)** Filtered orientation error on *x*-axis during the 4 g experiment. See Figure 5 for line interpretation. **(D)** Filtered orientation error on *y*-axis during the 4 g experiment. See Figure 5 for lines interpretation. **(E)** Filtered orientation error on *z*-axis during 4 g experiment. See Figure 5 for lines interpretation. **(F)** Continuation of **(E)**. The green vertical line indicates when the system is temporarily powered off.

**FIGURE 7:**
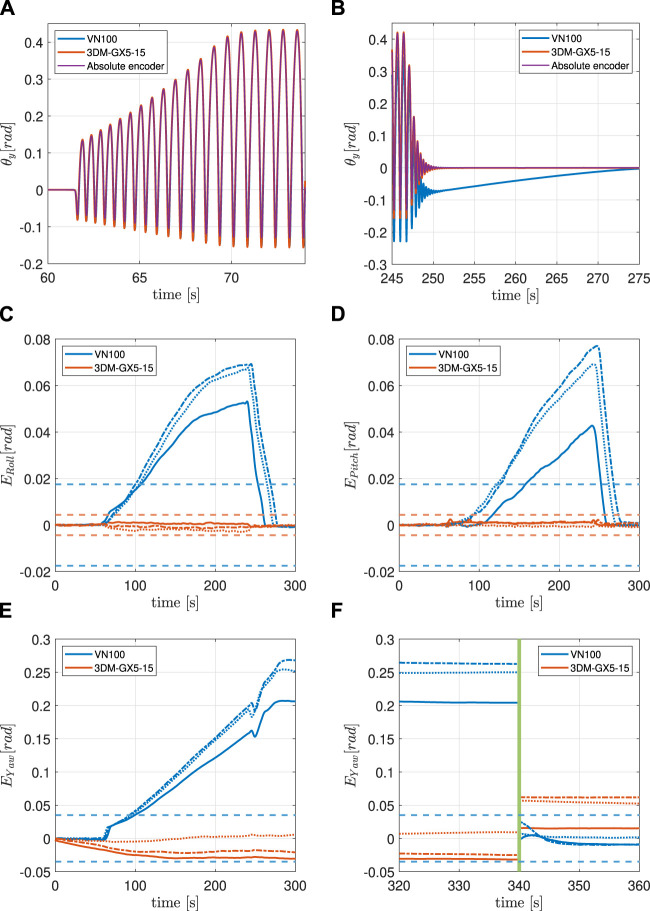
**(A)** IMU and encoder synchronized pitch measurements at the beginning of the 8 g experiment. **(B)** IMU and encoder synchronized pitch measurements at the end of the 8 g experiment. **(C)** Filtered orientation error on *x*-axis during the 8 g experiment. See Figure 5 for lines interpretation. **(D)** Filtered orientation error on *y*-axis during the 8 g experiment. See Figure 5 for lines interpretation. **(E)** iltered orientation error on *z*-axis during the 8 g experiment. See Figure 5 for lines interpretation. **(F)** Continuation of **(E)** The green vertical line indicates when the system is temporarily powered off.

**FIGURE 8:**
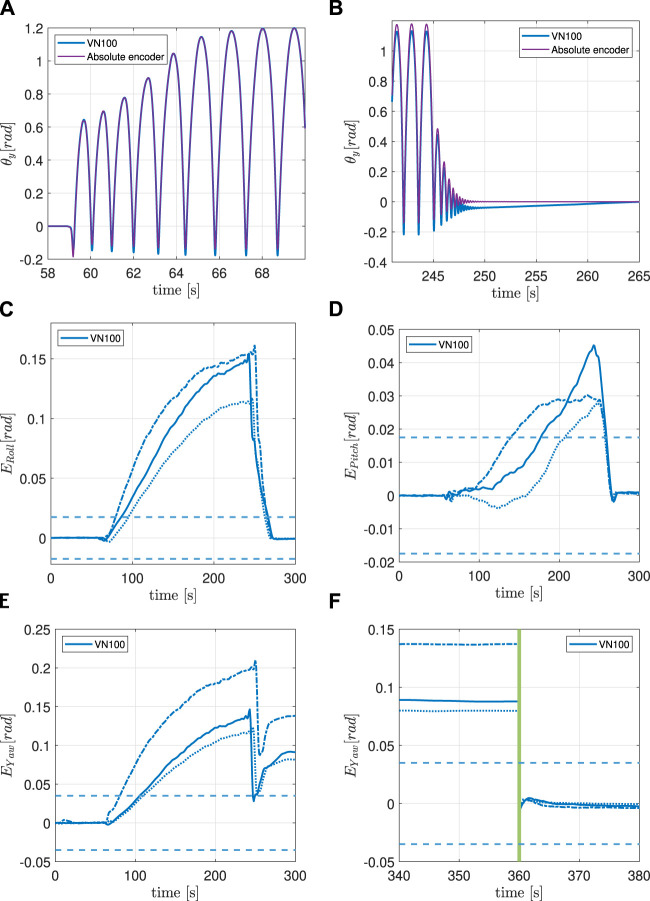
**(A)**IMU and encoder synchronized pitch measurements at the beginning of the 16 g experiment. **(B)** IMU and encoder synchronized pitch measurements at the end of the 16 g experiment. **(C)** Filtered orientation error on *x*-axis during 16 g experiment. See Figure 5 for lines interpretation. **(D)** Filtered orientation error on *y*-axis during 16 g experiment. See Figure 5 for lines interpretation. **(E)** Filtered orientation error on *z*-axis during 16 g experiment. See Figure 5 for lines interpretation. **(F)** Continuation of **(E)**. The green vertical line indicates when the system is temporarily powered off.

Figure 5 contains a general description of all the lines included in the error graphs (Figures 6C–F–F, 7C–F–F, 8C–F–F). For each experiment, the behaviour of the Roll, Pitch and Yaw angles is analyzed separately.

### 5.1 Experiment 1: 4 g maximum linear acceleration

In this experiment, the maximum value of linear acceleration along the *z*-axis is approximately 4 g and is close to but strictly below the saturation limit of the BNO055 (Table 2). All three IMUs have been tested under the same conditions. Figure 6A clearly shows how the three IMUs signals are all aligned with the encoder at the beginning of the experiment. Figure 6B displays the drift that both the VN100 and the BNO055 have accumulated during the motion, with the latter being higher in magnitude than the former. The 3DM-GX5-15 instead proved to be consistent in the estimation of the Pitch angle throughout the duration of the experiment.

#### 5.1.1 Roll

Surprisingly, the BNO055 performs better than the VN100 in the estimation of the Roll angle which shows some not-negligible drift effects in the estimation of the orientation. It takes the VN100 around 80 s to exceed the datasheet error (i.e., go out of spec) after the motion starts, and 20 s to return inside the interval once the bouncing stops. The 3DM-GX5-15, proved to be robust to this kind of motion.

#### 5.1.2 Pitch

The VN100 shows a small drift in the measurement of the Pitch angle only in one out of the three trials, with *E*_Pitch_ going slightly outside the accuracy range. The BNO055 absolute orientation estimation drifts significantly, making its values unreliable and impossible to be used in these circumstances. Also in this case the 3DM-GX5-15 gives reliable results, never going out of spec.

#### 5.1.3 Yaw

The VN100 measures accurately the Yaw angle. As a matter of fact, the *E*_Yaw_ is never outside the accuracy interval. Both the 3DM-GX5-15 and the BNO055 show a drift in the estimation of the Yaw angle. It can be observed that in both cases, the *E*_Yaw_ increases during the bouncing and it never returns to zero, even when the motion ceases. This behaviour we think is due to the fact that these two IMUs cannot rely on the compensation of the magnetometer, the 3DM-GX5-15 does not have it while the BNO055 cannot calibrate it.

#### 5.1.4 Power cycle

All of the three IMUs have been powered off and on (power cycle) after every trial to see the effects on the estimation of the Yaw angle. The VN100 is still inside the accuracy range as expected. The 3DM-GX5-15 starts evaluating again the Yaw angle with an offset different from the initial one due to the lack of magnetometer data. The *E*_Yaw_ for this IMU never returns to the initial value. The BNO055, instead, restarts from the initial value, showing an error almost null.

### 5.2 Experiment 2: 8 g maximum linear acceleration

The following experiment has the intent of testing the VN100 together with the 3DM-GX5-15 with a linear acceleration as close as possible to 8 g, which is the acceleration saturation limit of the 3DM-GX5-15 (Table 2). Also in this case, the two IMUs have the *y*-axis aligned with the solenoid and the signals are synchronized, see Figure 7A. Like in the previous experiment, the 3DM-GX5-15 proved to be a better estimator of *θ*_*y*_ than the VN100 which drifts significantly during the motion, Figure 7B.

#### 5.2.1 Roll and pitch

The VN100 shows evident drift in the estimation of both the Roll and the Pitch angles. It takes approximately 40 s for the *E*_Roll_ to exceed the accuracy limit, and 55 s for the *E*_Pitch_. Both these errors return inside the accuracy interval around 25 s after the motion ceases. In both cases the 3DM-GX5-15 shows a negligible error.

#### 5.2.2 Yaw

Interesting results are shown in the estimation of the Yaw angle where the VN100 shows a significant and persistent drift. *E*_Yaw_ crosses the accuracy threshold after 40 s and then persists also after the bouncing stops, with a magnitude of around 20°. On the other hand, the 3DM-GX5-15 shows a small drift in magnitude, but with sign not constant through the trials (it is positive in one trial and negative in the other two).

#### 5.2.3 Power cycle

The Yaw error of the VN100 does not return inside the accuracy interval after the bouncing stops. It requires a power cycle to make *E*_Yaw_ return between the boundaries of such interval. The power cycle forces the 3DM-GX5-15 to restart estimating the Yaw angle from a value which is different both from the initial one and the one before the power cycle.

### 5.3 Experiment 3: 16 g maximum linear acceleration

The last experiment analyzes the behaviour of the VN100 near its acceleration saturation limit at 16 g (Table 2). Figure 8A shows that the IMU’s measurement is aligned with the encoder’s one at the beginning of the motion, while Figure 8B clearly shows that the two signals are not aligned anymore when the motion stops.

#### 5.3.1 Roll and pitch

The drift on the Roll and Pitch angles is significant and not negligible, but it does not affect the absolute estimation once the bouncing finishes; both *E*_Roll_ and *E*_Pitch_ return inside the accuracy interval after 25 s. *E*_Roll_ crosses the accuracy threshold after 20 s from the start of the motion, while *E*_Pitch_ requires 70–130 s to reach the limit, depending on the trial.

#### 5.3.2 Yaw

On the other hand, the drift on the estimation of the Yaw angle is significant, it exceeds the threshold after 20—40 s (depending on the trial) and persists once the IMU returns to the initial rest position. *E*_Yaw_ varies from 5 to 10°, depending on the trial.

#### 5.3.3 Power cycle

Like in the 8 g experiment, a power cycle proved to be necessary to bring the *E*_Yaw_ back inside the accuracy interval.

## 6 Discussion

The IMU that displayed the worst performance in the 4 g experiment is the BNO055, which was the cheapest out of the three IMUs. The drift in the estimation of the Yaw angle was more than 4° and the BNO055 was the only IMU that could not recover the initial orientation after the bouncing motion had stopped. A simple power cycle proved to be partially effective: it brings back *E*_Yaw_ to its original value but it leaves the IMU not calibrated.

The 3DM-GX5-15 achieved the best performance in all the conditions where it has been tested. It is the only evaluated IMU that does not have a drift in estimating the Roll and Pitch angles both in the 4 and 8 g experiments. The drift on the Yaw angle, instead, is due to the absence of the magnetometer and cannot be compensated. In this case a power cycle proved to be ineffective.

The VN100 is the only IMU tested in all three experiments. It presents a drift in the estimation of the Roll angle in every experiment, and in the 4 g experiment it behaves worse than the BNO055. In all the experiments, both *E*_Roll_ and *E*_Pitch_ return inside the accuracy range once the motion stops. During the 4 g experiment the IMU does not show any drift in the Yaw angle estimation. Such behaviour is not present in the 8 g and 16 g experiments, where a power cycle turned out to be necessary to bring back the *E*_Yaw_ into the accuracy range.

## 7 Conclusion

This work is intended to give the reader a better understanding of the behaviour of MEMS IMUs in an environment for which they have not been specifically designed. The experiments reported in this paper investigated the orientation estimation performance of a selection of IMUs, subject to continuous low-intensity impacts, which aim to reproduce impacts experienced during hopping or running. According to the results, all three IMUs suffered a certain amount of drift during the experiments.

The continuous bouncing of the specimen resembles the motion of a bipedal or quadruped robot running. Therefore using these MEMS IMUs as a balancing reference point may lead to unexpected results, making it difficult to control the robot. The evident drift in the absolute orientation estimation cannot be left out in the design process of the control system, which has to be robust enough either to withstand or to compensate for it.

The natural extension of this work would be both to test more IMUs and to implement techniques to compensate for the drifts on all the axes. Alternatively, having this knowledge, the simplest thing to do would be for the robot to rest until the drift gets eliminated, similar to humans that stop when they are disoriented.

## Data Availability

The raw data supporting the conclusions of this article will be made available by the authors, without undue reservation.

## Footnotes

1
https://www.bostondynamics.com/atlas, accessed April 2023.

2
https://www.magnet-schultz.com/en/rotary-solenoids/proportional-rotary-solenoids-type-g-dr/, accessed April 2023.

3
https://www.pololu.com/product/2995, accessed April 2023.

4
https://www.rls.si/eng/, accessed April 2023.

5
https://www.ni.com/it-it/support/model.sbrio-9637.html, accessed April 2023.

6
https://www.vectornav.com/products, accessed April 2023.

7
https://www.microstrain.com/inertial-sensors/3dm-gx5-15, accessed April 2023.

8
https://www.microstrain.com/software/sensorconnect, accessed April 2023.

9
https://www.bosch-sensortec.com/products/smart-sensors/bno055/, accessed April 2023.

10
https://www.dfrobot.com/product-1793.html, accessed April 2023.

11
https://learn.adafruit.com/adafruit-bno055-absolute-orientation-sensor, accessed April 2023.

12
https://store.arduino.cc/products/arduino-uno-rev3, accessed April 2023.

## References

[B1] Abdulrahim K. Hide C. Moore T. Hill C. (2010). “Aiding mems imu with building heading for indoor pedestrian navigation,” in 2010 ubiquitous positioning indoor navigation and location based service, 1–6. 10.1109/UPINLBS.2010.5653986

[B2] Allione F. Gkikakis A. E. Featherstone R. (2022). “Experimental demonstration of a general balancing controller on an untethered planar inverted double pendulum,” in 2022 IEEE/RSJ International Conference on Intelligent Robots and Systems (IROS), Kyoto, Japan, 23-27 Oct. 2022 (IEEE), 8292–8297. 10.1109/IROS47612.2022.9981380

[B3] Allione F. Singh B. R. P. Gkikakis A. E. Featherstone R. (2021). “Mechanical shock testing of incremental and absolute position encoders,” in 2021 IEEE Int. Conf. on Advanced Robotics (ICAR), Ljubljana, Slovenia, 6-10 Dec. 2021 (IEEE), 52–57. 10.1109/ICAR53236.2021.9659349

[B4] Arlotti J. S. Carroll W. O. Afifi Y. Talegaonkar P. Albuquerque L. Ball J. E. (2022). Benefits of imu-based wearables in sports medicine: narrative review. Int. J. Kinesiol. Sports Sci. 10, 36–43. 10.7575/aiac.ijkss.v.10n.1p.36

[B5] Bhattacharya A. McCutcheon E. Shvartz E. Greenleaf J. (1980). Body acceleration distribution and o2 uptake in humans during running and jumping. J. Appl. physiology Respir. Environ. Exerc. physiology 49, 881–887. 10.1152/jappl.1980.49.5.881 7429911

[B6] Cardarelli S. Mengarelli A. Tigrini A. Strazza A. Di Nardo F. Fioretti S. (2020). Single imu displacement and orientation estimation of human center of mass: A magnetometer-free approach. IEEE Trans. Instrum. Meas. 69, 5629–5639. 10.1109/TIM.2019.2962295

[B7] Gkikakis A. E. (2021). Mechanism and behaviour Co-optimisation of high performance mobile robots. Ph.D. thesis, University of Genoa. 10.15167/gkikakis-antonios-emmanouil_phd2021-04-21

[B8] Hutter M. Gehring C. Jud D. Lauber A. Bellicoso C. D. Tsounis V. (2016). “Anymal - a highly mobile and dynamic quadrupedal robot,” in 2016 IEEE/RSJ International Conference on Intelligent Robots and Systems (IROS), Daejeon, Korea (South), 9-14 Oct. 2016, 38–44. 10.1109/IROS.2016.7758092

[B9] Katz B. Carlo J. D. Kim S. (2019). “Mini cheetah: A platform for pushing the limits of dynamic quadruped control,” in 2019 International Conference on Robotics and Automation (ICRA), Montreal, QC, Canada, 20-24 May 2019 (IEEE), 6295–6301. 10.1109/ICRA.2019.8793865

[B10] Koga C. Miyase K. Tokui M. (2020). “Analyzing running form with acceleration sensor,” in In 2020 IEEE International Conference on Consumer Electronics (ICCE), Las Vegas, NV, USA, 4-6 Jan. 2020 (IEEE), 1–5. 10.1109/ICCE46568.2020.9043124

[B11] Kuindersma S. Deits R. Fallon M. Valenzuela A. Dai H. Permenter F. (2015). Optimization-based locomotion planning, estimation, and control design for the atlas humanoid robot. Aut. Robots 40, 429–455. 10.1007/s10514-015-9479-3

[B12] Li M. Jiang Z. Wang P. Sun L. Sam Ge S. (2014). Control of a quadruped robot with bionic springy legs in trotting gait. J. Bionic Eng. 11, 188–198. 10.1016/S1672-6529(14)60043-3

[B13] Liang J. Duan H. Li J. Sun H. Sha X. Zhao Y. (2018). “Accurate estimation of gait altitude using one wearable imu sensor,” in 2018 IEEE 1st International Conference on Micro/Nano Sensors for AI, Healthcare, and Robotics (NSENS), Shenzhen, China, 5-7 Dec. 2018 (IEEE), 64–67. 10.1109/NSENS.2018.8713562

[B14] Qi W. Aliverti A. (2020). A multimodal wearable system for continuous and real-time breathing pattern monitoring during daily activity. IEEE J. Biomed. Health Inf. 24, 2199–2207. 10.1109/JBHI.2019.2963048 31902783

[B15] Raibert M. H. (1986). Legged robots that balance. MIT press.

[B16] Safaeifar A. Nahvi A. (2015). “Drift cancellation of an orientation tracker for a virtual reality head-mounted display,” in 2015 3rd RSI International Conference on Robotics and Mechatronics (ICROM), Tehran, Iran, 7-9 Oct. 2015 (IEEE), 296–301. 10.1109/ICRoM.2015.7367800

[B17] Semini C. Barasuol V. Goldsmith J. Frigerio M. Focchi M. Gao Y. (2017). Design of the hydraulically actuated, torque-controlled quadruped robot hyq2max. IEEE/ASME Trans. Mechatronics 22, 635–646. 10.1109/TMECH.2016.2616284

[B18] Semini C. Tsagarakis N. G. Guglielmino E. Focchi M. Cannella F. Caldwell D. G. (2011). Design of hyq – A hydraulically and electrically actuated quadruped robot. Proc. Institution Mech. Eng. Part I J. Syst. Control Eng. 225, 831–849. 10.1177/0959651811402275

[B19] Tan T. Strout Z. A. Xia H. Orban M. Shull P. B. (2021). Magnetometer-free, imu-based foot progression angle estimation for real-life walking conditions. IEEE Trans. Neural Syst. Rehabilitation Eng. 29, 282–289. 10.1109/TNSRE.2020.3047402 33360997

[B20] Yang D. Huang J. Tu X. Ding G. Shen T. Xiao X. (2018). A wearable activity recognition device using air-pressure and imu sensors. IEEE access 7, 6611–6621. 10.1109/access.2018.2890004

[B21] Yim J. K. Singh B. R. P. Wang E. K. Featherstone R. Fearing R. S. (2020). Precision robotic leaping and landing using stance-phase balance. IEEE RA-L 5, 3422–3429. 10.1109/lra.2020.2976597

[B22] Zihajehzadeh S. Loh D. Lee M. Hoskinson R. Park E. (2014). “A cascaded two-step kalman filter for estimation of human body segment orientation using mems-imu,” in 2014 36th Annual International Conference of the IEEE Engineering in Medicine and Biology Society, Chicago, IL, USA, 26-30 Aug. 2014, 6270–6273. 10.1109/EMBC.2014.6945062 25571430

[B23] Zihajehzadeh S. Loh D. Lee T. J. Hoskinson R. Park E. J. (2015). A cascaded kalman filter-based gps/mems-imu integration for sports applications. Measurement 73, 200–210. 10.1016/j.measurement.2015.05.023

